# Physical properties and field-induced metamagnetic transitions in UAu_0.8_Sb_2_

**DOI:** 10.1038/s41598-018-26314-7

**Published:** 2018-05-18

**Authors:** Wen Zhang, Chunyu Guo, Donghua Xie, Michael Smidman, Bingfeng Hu, Yuanhua Xia, Yi Liu, Shiyong Tan, Wei Feng, Xiegang Zhu, Yun Zhang, Qunqing Hao, Lizhu Luo, Huiqiu Yuan, Xinchun Lai

**Affiliations:** 1 Science and Technology on Surface Physics and Chemistry Laboratory, Mianyang, 621908 China; 2 Center for Correlated Matter and Department of Physics, Zhejiang University, Hangzhou, 310058 China; 3 Key laboratory of neutron physics, Institute of nuclear physics and chemistry, China academy of engineering physics, Mianyang, 621999 China

## Abstract

We have successfully synthesized single crystals of UAu_0.8_Sb_2_ using a flux method and present a comprehensive study of its physical properties by measuring the magnetic susceptibility, electrical resistivity and specific heat. Evidence for at least three magnetic phases is observed in the field-temperature phase diagram of UAu_0.8_Sb_2_. In zero field, the system undergoes an antiferromagnetic transition at 71 K, and upon further cooling it passes through another antiferromagnetic phase with a ferromagnetic component, before reaching a ferromagnetic ground state. A complex magnetic field-temperature phase diagram is obtained for fields along the easy c-axis, where the antiferromagnetic order eventually becomes polarized upon applying a magnetic field.

## Introduction

In recent decades, the heavy-fermion behavior and magnetic order of actinide-based intermetallics has been the focus of much research^1,2^. In many U-based compounds, the Kondo effect can quench the localized magnetic moments and lead to a non-magnetically-ordered heavy-fermion state while another competing interaction, the Ruderman-Kittel-Kasuya-Yosida (RKKY) exchange interaction couples localized spins and favors long-range magnetic order. The competition between these effects can lead to a variety of interesting physical phenomena, such as heavy-fermion behavior and unconventional superconductivity in UPt_3_^3,4^, hidden order in URu_2_Si_2_^5–7^, ferromagnetism in UAuBi_2_^8^, and the coexistence of superconductivity and ferromagnetism in URhGe^9^ and UCoGe^10^. The “112” uranium-based ternary compounds U*TX*_2_ (*T* = transition metal, *X* = pnictogen) are a good platform to study the competing interactions, which can be tuned by varying the transition metal or the site occupancies^11–16^. The U*TX*_2_ materials crystallize in the tetragonal HfCuSi_2_-type structure (space group *P4/nmm*, No. 129), where layers of *T*, U-*X* and *X* are stacked along the *c*-axis, leading to strongly anisotropic properties in many of these compounds^11,13^. A variety of magnetic properties in this family have been found, where the compounds with *T* = Co, Cu, Ag, and Au are reported to display ferromagnetic order, while those with *T* = Ni, Ru, and Pd are antiferromagnetic^11,12^. While most studies have been on polycrystalline samples, single crystals of members have been grown using Sb flux, which generally have partial occupancies at the *T* site. The U*T*_*1-x*_Sb_2_ compounds UNi_0.5_Sb_2_^13,14^, UCo_0.5_Sb_2_^15^, UCu_0.9_Sb_2_^16^, and UPd_0.6_Sb_2_^17^ show some properties which differ from the corresponding materials with full occupancies. For example, UNi_0.5_Sb_2_ has been found to display a different magnetic ordering temperature, with two further anomalies at lower temperatures^14^. As such it is important to examine the magnetic properties of single crystals of partially occupied compounds in the U*T*_*1-x*_Sb_2_ series, which may help understand how the complex magnetism may be tuned.

Here we report the synthesis of UAu_0.8_Sb_2_ single crystals using a self-flux method and study the physical properties using electrical resistivity, magnetic susceptibility and heat capacity. Our results reveal a possible antiferromagnetic transition (AFM1) at *T*_*N*_ = 71 K, another antiferromagnetic transition (AFM2) at 34 K and a ferromagnetic transition at 10 K, all with the easy axis of magnetization along the *c* axis. From detailed measurements with fields applied along the easy direction, we map the field-temperature phase diagram.

## Results

### Crystal structure and physical properties

**Figure 1:**
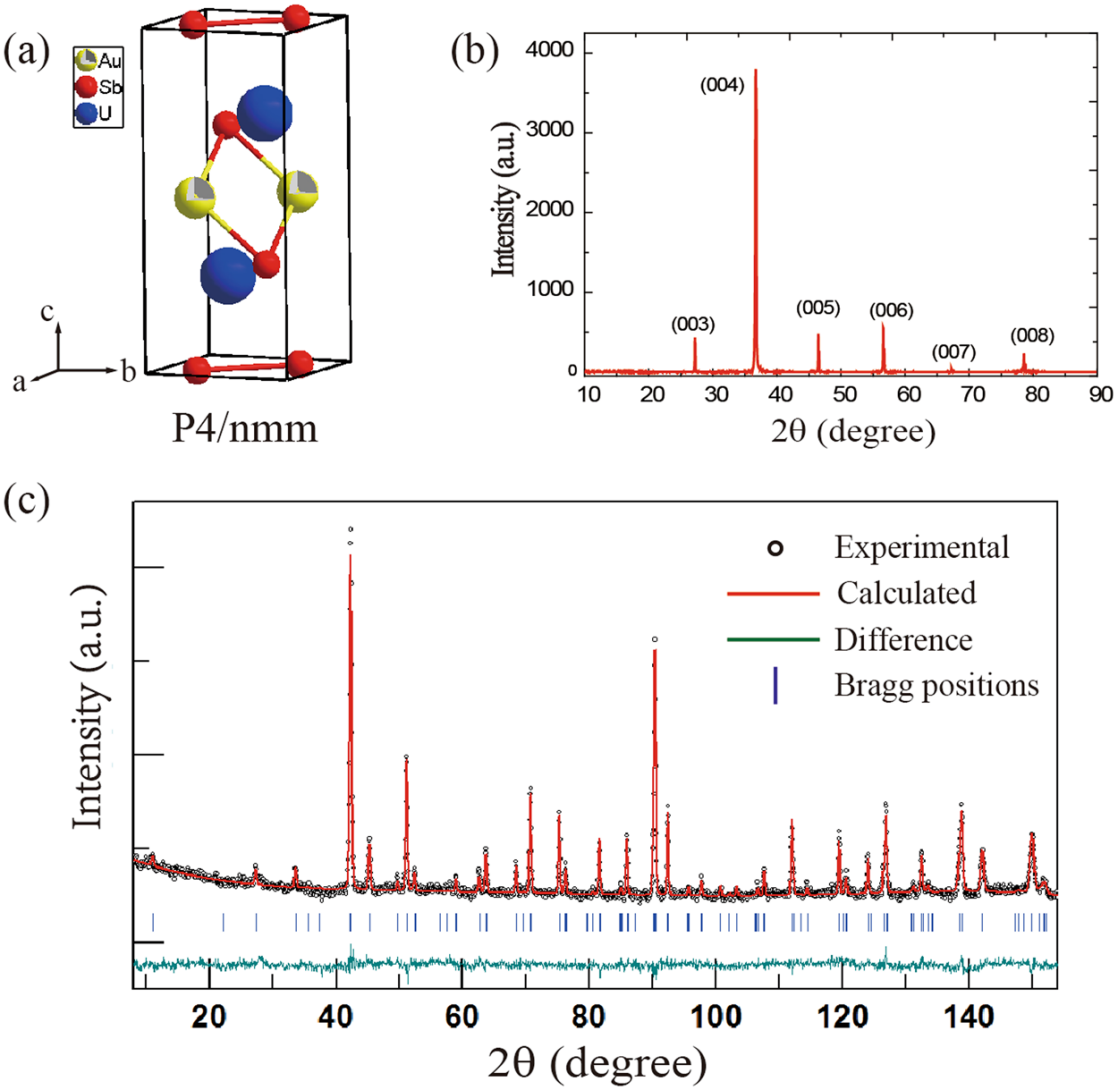
(**a**) Crystal structure of UAu_0.8_Sb_2._ (**b**) X-ray diffraction pattern of a UAu_0.8_Sb_2_ single crystal. (**c**) Powder neutron diffraction pattern of UAu_0.8_Sb_2_ performed at room temperature, where the calculated results from the structural refinement are also displayed, as well as the difference between the data and the calculations.

Figure 1 displays the crystal structure, x-ray diffraction (XRD) and neutron diffraction patterns of UAu_0.8_Sb_2_ at room temperature. The XRD measurements in Fig. 1(b) were performed on a single crystal sample, and the peaks can all be accounted for by the (00 *l*) reflections, indicating that the *c*-axis is perpendicular to the large face of the crystal. The neutron diffraction pattern indicates that the sample is single phase, and can be refined with the tetragonal HfCuSi_2_-type structure^18^. Table 1 shows the results of the refinement, while the atomic coordinates and occupancy factors are provided in Table 2. The refined lattice parameters and unit cell volume are *a* = 0.436320(6) nm, *c* = 0.978574(3) nm and *V* = 0.1862963(6) nm^3^, which are smaller than those reported for UAuSb_2_ (*a* = 0.4375(1) nm, *c* = 0.9831(2) nm)^19^. Upon refining the site occupancies keeping those of the U site fixed to one, the Sb sites are very close to being fully occupied, but the occupancy of the Au site is 0.792(2).

**Table 1:** Results from the structural refinements of the powder neutron diffraction measurements of UAu_0.8_Sb_2_.

Compound	UAu_0.8_Sb_2_
Crystal system, space group	tetragonal, *P4/nmm*
Cell parameters (nm)	a = 0.436320(6), c = 0.978574(3)
Volume (nm^3^)	0.1862963(6)
**Rietveld reliability factors**	
*R*_p_	4.14
*R*_*wp*_	5.26
χ^2^	1.73

The lattice parameters as well as the reliability factors for the refinement are also displayed.

**Table 2:** Crystallographic parameters and site occupancies (s.o.f) for the refinements of the powder neutron diffraction measurements of UAu_0.8_Sb_2_.

Atom	Wyckoff position	x	y	z	s.o.f.
U	2c	0.25	0.25	0.24378(40)	1
Sb1	2c	0.25	0.25	0.68246(66)	0.96(2)
Sb2	2a	0.75	0.25	0.00	0.98(1)
Au	2b	0.75	0.25	0.50	0.792(2)

**Figure 2:**
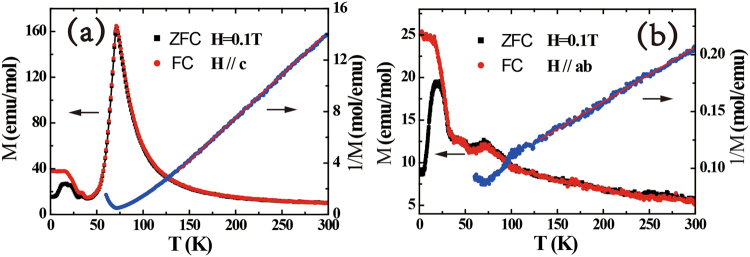
Temperature dependence of the dc-magnetization *M*(*T*) (left-hand scale) and inverse dc-magnetization (right-hand scale) measured in an applied magnetic field of 0.1 T parallel to (**a**) the *c*-axis, and (**b**) the *ab*-plane. Both zero-field cooled (ZFC) and field-cooled (FC) measurements are displayed. The solid lines display fits to the inverse magnetization using the Curie-Weiss expression (Eq. 1).

Figure 2(a,b) display the dc magnetization *M*(*T*) measured in a magnetic field of 0.1 T applied parallel to the *c*-axis and *ab* plane, respectively. The magnetization for fields applied in the *ab* plane is much smaller than along the *c*-axis, indicating significant magnetic anisotropy in UAu_0.8_Sb_2_, where the *c*-axis is the easy magnetization direction. Above 140 K, *M*(*T*) can be fitted using a modified Curie-Weiss expression:

1 $${\rm{\chi }}={{\rm{\chi }}}_{0}+\frac{{\rm{C}}}{{\rm{T}}-{{\rm{\theta }}}_{{\rm{p}}}},$$

where ${\rm{C}}={{\rm{N}}{\rm{\mu }}}_{{\rm{B}}}^{2}{{\rm{\mu }}}_{{\rm{eff}}}^{2}/3{{\rm{k}}}_{{\rm{B}}}$, giving an effective moment of μ_eff_ = 3.47 μ_B_/U and a Curie-Weiss temperature of θ_p_ = 84.8 K for fields parallel to [001], and μ_eff_ = 3.07 μ_B_/U and θ_p_ = −9.7 K for fields in the *ab-*plane. The μ_eff_ values are reduced compared to the effective magnetic moment of the free U^4+^ ions and U^3+^ ions (µ_eff_ = 3.58μ_B_/U and 3.62 μ_B_/U, respectively). The value of θ_p_ is positive along the *c*-axis and negative in the *ab* plane, indicating the presence of both ferromagnetic and antiferromagnetic exchange interactions. The magnetization increases with decreasing temperature before reaching a maximum and abruptly dropping, which likely corresponds to a magnetic transition at *T*_*N*_ = 71 K. At lower temperatures, splitting of the zero-field cooled (ZFC) and field-cooled (FC) curves can be seen at around 38 K for fields parallel to [001], along with another small peak at 34 K. This suggests the onset of a ferromagnetic component and a transition to a different magnetic state.

**Figure 3:**
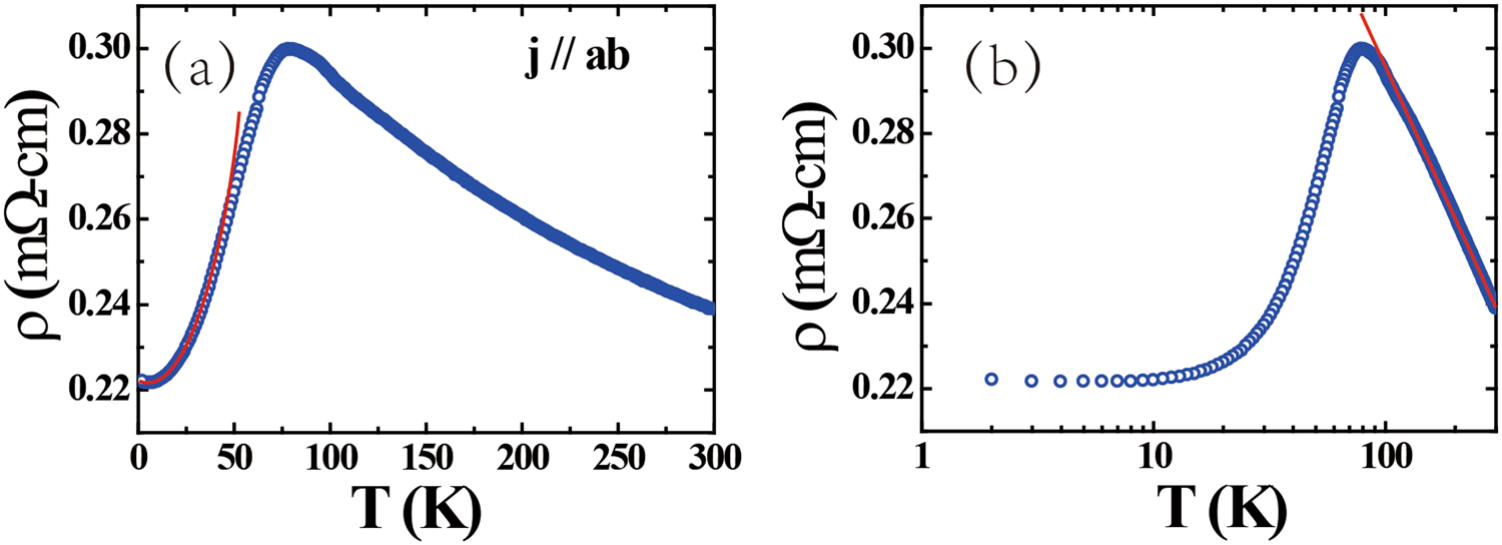
(**a**) Temperature dependence of the resistivity, where the solid red line represents a fit to Eq. 2. (**b**) Resistivity on a logarithmic temperature scale, where the solid red line represents a fit to Eq. 3.

Figure 3(a) shows the temperature dependence of the resistivity. The resistivity increases with decreasing temperature, reaching maximum at about 78 K, which is a little higher than the *T*_*N*_ value derived from *M*(*T*), before dropping at lower temperatures. As displayed in the main panel, below 40 K the temperature dependence of the resistivity can be fitted using the expression^11,16^.

2 $${\rm{\rho }}({\rm{T}})={\rho }_{0}+A{T}^{2}\exp (-\frac{{\rm{\Delta }}}{T}),$$

where *ρ*_0_ is the residual resistivity and the second term describes scattering of the conduction electrons by spin-wave excitations with an energy gap Δ. The fitted values are *ρ*_0_ = 221.7 μΩ cm, *A* = 0.0257 μΩ cm/K^2^ and Δ = 16 K. In the paramagnetic state, above 100 K, the resistivity follows logarithmic behavior which may be due to Kondo scattering following

3 $$\rho (T)=\rho ^{\prime} +-\,{c}_{k}lnT,$$

where *ρ*′ is a temperature independent term. As shown in Fig. 3(b), this expression can describe the higher temperature data, indicating the presence of significant Kondo scattering.

**Figure 4:**
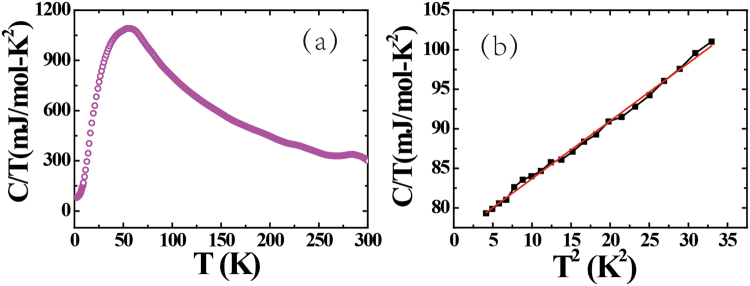
(**a**) Temperature dependence of the specific heat as *C*/*T*. (**b**) The low-temperature *C/T* as a function of *T*^2^, where the solid red line displays a linear fit to the data.

Figure 4(a) shows the temperature dependence of the specific heat, where a kink in *C/T* is observed at 78 K, which likely corresponds to the magnetic transition. As shown in Fig. 4(b), at low temperatures C/T ~ T^2^, and from extrapolating to zero temperature a value of the electronic specific heat coefficient γ of 76 mJ/mol∙K^2^ is estimated, indicating that UAu_0.8_Sb_2_ has moderately enhanced correlations due to the Kondo effect.

### Field dependent measurements

**Figure 5:**
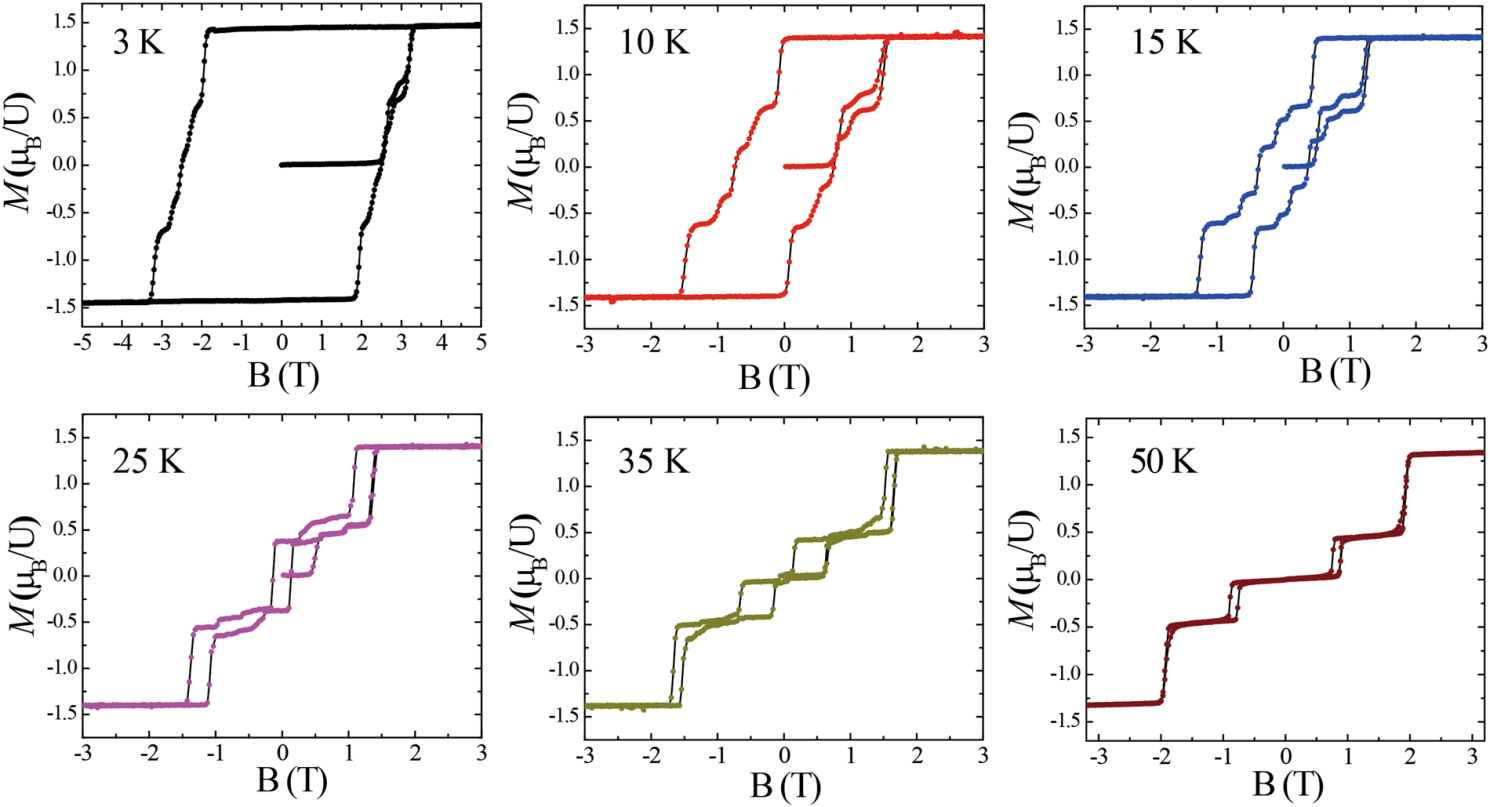
The magnetization as a function of applied magnetic field parallel to the *c* axis at different temperatures.

Figure 5 shows the field dependence of the magnetization for fields applied parallel to the *c* axis at different temperatures. At 50 K, the zero-field magnetization is nearly zero for both the up-sweep and down-sweep processes with little hysteresis, indicating that at this temperature the system orders antiferromagnetically. Two metamagnetic transitions are observed with increasing magnetic field, one at around 0.9 T and another at 1.9 T, above which the magnetization changes little suggesting that this corresponds to a spin-polarized phase with a saturated moment of around 1.35 μ_B_/U. The plateau inbetween 0.9 and 1.9 T has a magnetization of about one third of the saturated value, suggesting that this corresponds to a field-induced antiferromagnetic phase which also has an FM component (AFM2). Such a magnetization plateau could arise if the spins are arranged periodically in a collinear ‘up-up-down’ arrangement, which yields a net magnetization of one third of that of the ferromagnetic state. It can be seen that at 35 K, the transition to the AFM2 phase with the one third magnetization moves to lower fields. The hysteresis at both metamagnetic transitions significantly increases, and a small hysteresis develops around zero-field, which is consistent with the splitting of the ZFC and FC curves at a slightly higher temperature in Fig. 2(a). At 25 and 15 K, there is now a clear hysteresis loop about zero-field, where the remanent magnetization is nearly one-third of the saturated value, indicating that the AFM2 state with both AFM and FM components is present at zero-field in this temperature range. A number of additional steps in the magnetization can also be detected below the metamagnetic transition to the field-induced FM state. While those in the upsweep curves (from 0 to ±9 T) may arise from the alignment of magnetic domains, at least one step can be resolved in the down-sweep measurements. This may either signify an additional change of magnetic structure, or the coexistence of the AFM2 state with small regions of either the AFM or FM phases.

At lower temperatures still, as shown for example by the curve at 3 K, it can be seen that upon sweeping down from high fields to zero-field, the magnetization remains very near the high-field saturated value, giving strong evidence for a ferromagnetic ground state. The magnetization changes little as the field is reduced into the negative field range, before the sign of the magnetization switches. It can be seen that the magnetization undergoes a number of steps before reaching the saturated negative value, which is likely related to the domain reorientation processes. It is noted that if the field sweep-rate is increased these steps are found to disappear.

**Figure 6:**
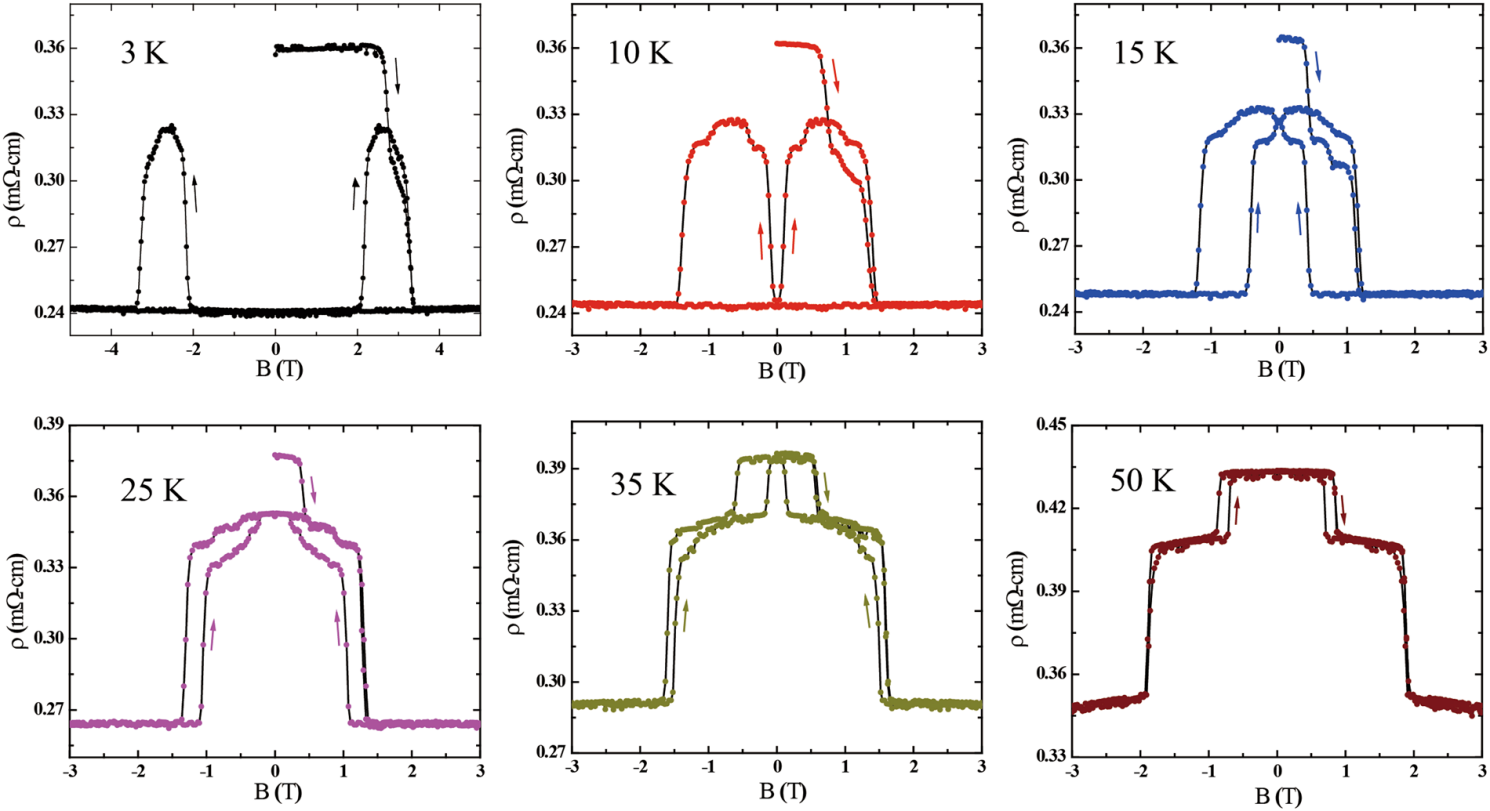
In-plane resistivity of UAu_0.8_Sb_2_ as a function of applied magnetic field along the *c* axis at different temperatures. The arrows indicate the direction of the changing field.

Figure 6 shows the in-plane resistivity of UAu_0.8_Sb_2_ as a function of applied magnetic field along the *c* axis at different temperatures. In agreement with the magnetization measurements, the data at 50 K display two jumps of the resistivity in field with little hysteresis. At both of these metamagnetic transitions there is a sharp drop of the resistivity with increasing field, which is consistent with the sudden transition to a magnetic phase with a larger ferromagnetic component. Meanwhile below 35 K, the hysteresis at the low-field metamagnetic transition again significantly increases. At 15 and 25 K where the magnetization indicates the AFM2 ground state, the in-field hysteresis in the magnetoresistance is observed over a broader field range, only disappearing when the moments align in the field-induced FM state. Meanwhile at lower temperatures, where the magnetization loops indicate a ferromagnetic ground state, the hysteresis in the magnetoresistance disappears at low fields and the hysteresis loops only open in the field region around the coercive field, where the ferromagnetic domains reorientate.

### Phase diagram

**Figure 7:**
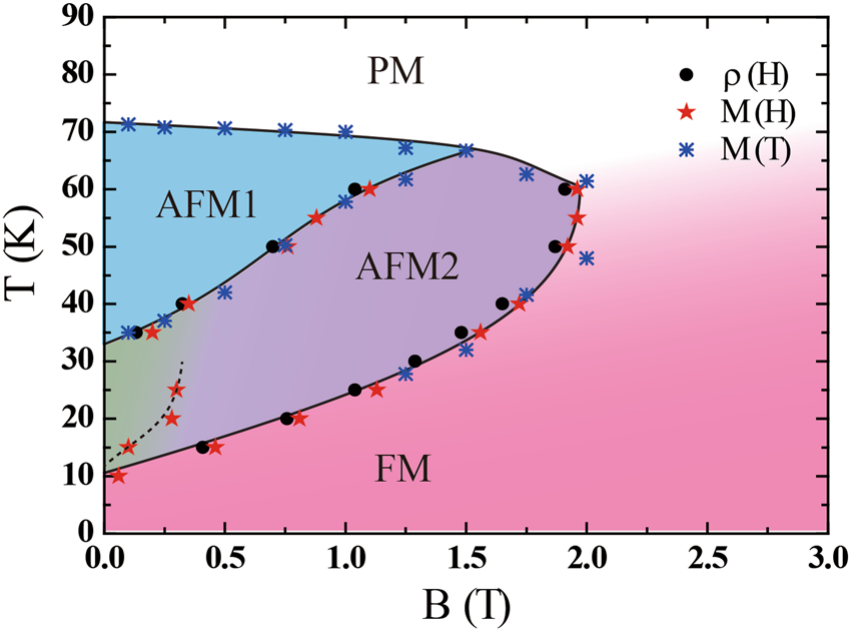
Field-temperature phase diagram derived from the resistivity and magnetization data, for an external field applied parallel to the *c* axis.

Figure 7 shows the field-temperature phase diagram derived from the magnetoresistance and magnetization data, when the external field is applied parallel to the *c* axis. For the field dependent quantities, the locations of the transitions were determined from the down-sweep measurements from high fields. It can be seen that upon cooling from high temperatures in zero field the system undergoes an antiferromagnetic transition to a phase labeled AFM1. Meanwhile upon increasing the field along the *c*-axis, the system changes to the AFM2 phase, which has both antiferromagnetic and ferromagnetic components. Since the net magnetization is around one third of the saturated value, this suggests a magnetic structure along the lines of an ‘up-up-down’ spin configuration. At higher fields still, the spins align in a field induced ferromagnetic state. Upon cooling further, the AFM1 phase is not found and in zero field the system is in the AFM2 state, where there is a finite remanent magnetization in zero field. The position of the additional magnetization steps, are marked by the dashed line. Meanwhile at low temperatures the ground state becomes ferromagnetic, as evidenced by the ferromagnetic-like hysteresis loops in the magnetization. To understand the nature of the magnetic phases and determine the magnetic structure, low temperature neutron diffraction measurements are highly desirable.

## Discussion

The U*T*_*1-x*_*X*_2_ materials present variety of magnetic properties including AFM and FM ground states^11–16^, which were suggested to arise due to the modification of the magnetic exchange interactions by hybridization between the *f* electrons and conduction electrons^11^. In most of these materials, only one magnetic ordering transition is reported, whereas others such as UCuBi_2_ display two AFM transitions^12^. The partially occupied UAu_0.8_Sb_2_ shares some similarities to both UAuSb_2_ and USb_2_. UAuSb_2_ likely undergoes two magnetic transitions, a ferromagnetic one at *T*_C_ = 31 K and a possible antiferromagnetic transition at *T*_N_ = 43 K^19^. Meanwhile USb_2_ undergoes an antiferromagnetic transition at 203 K, with no other magnetic transitions at lower temperature^20^. This suggests that the effect of Au doping into USb_2_ suppresses the antiferromagnetic interactions, reducing *T*_*N*_, but induces a ferromagnetic transition at lower temperature. On the other hand, since the partial occupancy leads to smaller lattice parameters, UAu_0.8_Sb_2_ may correspond to UAuSb_2_ at positive pressure, and therefore it is of interest to study the stoichiometric UAuSb_2_ under pressure to examine if the antiferromagnetism is enhanced accordingly.

In conclusion, we have successfully synthesized UAu_0.8_Sb_2_ single crystals and performed a detailed investigation of its crystal structure and physical properties. We find evidence for at least three magnetic phases, a high temperature antiferromagnetic phase, a phase with both ferromagnetic and antiferromagnetic components at intermediate temperatures, and a low temperature ferromagnetic phase. We also constructed a field-temperature phase diagram from measurements performed with a field applied along the easy direction.

## Methods

UAu_0.8_Sb_2_ single crystals were grown using a self-flux method^21^. U (99.9%), Au (99.999%) and Sb (99.9999%) were combined in an atomic ratio of 1:3.5:14 and placed in an alumina crucible. The crucible was sealed in an evacuated silica tube, heated up to 1150 °C and held at this temperature for 24 h before being cooled to 1050 °C over 1 h and slowly cooled down to 700 °C. The excess Sb flux was removed by centrifuging and plate like crystals were mechanically separated from the crucible. The typical dimensions of the crystals are about 4 × 4 × 2 mm^3^.

The x-ray diffraction measurements of the single crystal were performed using a PANalytical X′Pert Pro diffractometer (Cu K_α_-radiation). The neutron diffraction experiments for UAu_0.8_Sb_2_ powder were carried out using the high resolution neutron powder diffractometer (HRND) (λ = 0.1884 nm) at the China Mianyang Research Reactor (CMRR). The structural refinements were performed using the Fullprof software^22^. The measurements of the resistivity, magnetization and specific heat were performed using a Physical Property Measurement System (PPMS-9).
